# Gait and speech analysis for predicting progression in memory clinic: A two‐year longitudinal follow‐up

**DOI:** 10.1002/alz.088830

**Published:** 2025-01-03

**Authors:** Yi‐Chien Liu, Chia‐Ju Chou, Chia‐Ying Lee

**Affiliations:** ^1^ Medical school of Fu‐Jen University, Taipei Taiwan; ^2^ Cardinal Tien Hospital, New Taipei City Taiwan; ^3^ Institute of Linguistics, Academia Sinica, Taipei Taiwan

## Abstract

**Background:**

The clinical progression of AD exhibits significant heterogeneity among individuals. Early identification of individuals likely to experience disease progression holds paramount importance within the context of a memory clinic. In the present study, we endeavor to analyze gait and speech data as potential predictors of early AD progression.

**Methods:**

Between 2021 and 2023, we conducted a prospective recruitment of 99 participants from a memory clinic in Taiwan. All participants underwent comprehensive assessments, including amyloid PET scans, brain MRI scans, a battery of neuropsychological tests, and serum tests aimed at excluding reversible causes of cognitive impairment. Over a two‐year longitudinal follow‐up period, participants received diagnoses ranging from subjective cognitive decline (SCD), mild cognitive impairment (MCI), to mild dementia. Additionally, based on changes observed in the Clinical Dementia Rating Scale, Sum of Boxes (CDR‐SB) scores during the follow‐up period, participants were categorized into ‘progressor’ (CDR‐SB change≧1) and ‘non‐progressor’ groups. We collected speech and gait data through picture description tests and the Short Physical Performance Battery (SPPB). Speech data was manually transcribed and subsequently analyzed, focusing on 16 linguistic features.

**Results:**

In our cohort, 42.31% of individuals were categorized as progressors. The prevalence of amyloid positivity was notably higher among the progressors (84.09%) than in the non‐progressor group (29.63%). Our predictive modeling efforts involved logistic regression and the Receiver Operating Characteristic (ROC) curve analysis to forecast the progression of individuals within our dataset. Specifically, when we integrated gait speed and linguistic features as predictors, the Area Under the Curve (AUC) reached 0.8. In comparison, the AUC was 0.75 when we employed hippocampal volume as a predictor.

**Conclusion:**

Gait and speech data have the potential to serve as indicators of baseline cognitive status within a memory clinic setting. When combined, these datasets allow us to predict clinical progression with comparable accuracy to that achieved using neuroimaging techniques.

